# Imaging manifestations of papillary glioneuronal tumors

**DOI:** 10.1007/s10143-024-02393-1

**Published:** 2024-04-23

**Authors:** Xiaodan Du, Ying He, Feng Li, Xiaoye Wang, Xin Kong, Mei Ye, Xuzhu Chen

**Affiliations:** 1 Department of Medical Imaging, Zhuhai Hospital of Integrated Traditional Chinese and Western Medicine, Guangdong, 519000 China; 2 Department of Magnetic resonance, The People’s Hospital of Rizhao, Shandong, 276800 China; 3 Department of Radiology, Beijing Tiantan Hospital, Capital Medical University, Beijing, 100070 China

**Keywords:** Papillary glioneuronal tumor, Neuronal and mixed neuronal-glial tumors, Magnetic resonance imaging, Computed tomography

## Abstract

To analyse the imaging findings of papillary glioneuronal tumors (PGNTs), in order to improve the accuracy of preoperative diagnosis of this tumor. The clinical and imaging manifestations of 36 cases of PGNT confirmed by pathology were analyzed retrospectively. A total of 17 males and 19 females, averaging 22.47 (± 11.23) years. Initial symptoms included epilepsy in ten, headache in seven, and others in 19 cases. 97.2% (35/36) of the lesions were located in the supratentorial area, and 80.5% (29/36) in the intraventricular or deep white matter adjacent to the lateral ventricles. Twenty-four of the lesions (66.7%) were mixed cystic and solid, four (11.1%) were cystic with mural nodules, four (11.1%) were cystic, and four (11.1%) were solid. Four cases of PGNT of cystic imaging showed a “T2-FLAIR mismatch” sign. 69.4% (25/36) had septations. Nine lesions (25%) were accompanied by edema, and 9 (25%) of the mixed cystic and solid lesions were accompanied by hemorrhage. Among the 18 patients who underwent computed tomography (CT) or susceptibility-weighted imaging (SWI), nine had lesions with calcification. PGNTs mostly manifest as cystic mass with mural nodules or mixed cystic and solid mass in the white matter around the supratentorial ventricle, and the cystic part of the lesion is mostly accompanied by septations. Pure cystic lesions may exhibit the sign of “T2-FLAIR mismatch”. PGNT is rarely accompanied by edema but sometimes by calcification and hemorrhage. Patients often present with seizures, headaches, and mass effect symptoms.

## Introduction

Papillary glioneuronal tumors (PGNTs) were first named “pseudopapillary glioneurocytomas” by Komori et al. in 1996 [1]. In 2000, the World Health Organization (WHO) classified PGNT as a ganglioglioma variant [2], and the updated classification system in 2021 considered PGNTs a type among glioneuronal and neuronal tumors [3]. Although PGNTs were classified as Grade I tumors by the WHO in 2016, malignant or invasive PGNT cases have been continuously reported [4]. It is often misdiagnosed because of the rarity of PGNT and the lack of understanding of its imaging manifestations. Here, we summarize the imaging manifestations of 36 cases of PGNT and conduct a retrospective analysis in conjunction with the literature to improve the understanding of PGNT and the accuracy of preoperative diagnosis.

## Materials and methods

### Subjects

The Ethics Committee of our hospital approved this retrospective study, and the requirement for informed consent was waived.

The clinical and imaging data of 36 patients with PGNT confirmed by surgery and pathology between October 2011 and October 2022 were collected. Basic clinical data, including age, sex, major symptoms or signs, and methods of obtaining histopathological findings, were obtained by retrieving medical records. A total of 36 patients with PGNT were recruited based on the inclusion(PGNT confirmed by histopathology; MRI and/or CT were performed before surgery) and exclusion criteria (histopathology showed PGNT with other tumors and poor image quality).

### Imaging techniques

All 36 patients underwent plain MRI (T1WI and T2WI) and contrast-enhanced T1WI (CE-T1WI) scans. In addition, 20 underwent T2-FLAIR, 26 underwent DWI, 3 underwent SWI, and 16 underwent CT. Among them, one patient underwent SWI and CT simultaneously, while 13 patients underwent both CT and DWI examinations.

The scanning parameters of each MR machine model were 6–7 mm slice gap and 5–6 mm slice thickness. The contrast enhancement agent was gadolinium diethylenetriaminepentaacetic acid (Gd-DTPA) at a 0.1 mmol/kg dose via cubital vein mass injection. The scanning parameters are listed in Table 1. Sixteen patients underwent additional CT with scanning parameters of 120 kV, 413 mA, and a slice thickness of 4.5 mm.

**Table 1:** MRI scanning parameters

MRI scanners	T1WI		T2WI		CE-T1WI	
	TR (ms)	TE (ms)	TR (ms)	TE (ms)	TR (ms)	TE (ms)
GE NESIS-SIGNA	2115	29	4900	117	2031	19
GE Discovery MR750	2275	28	6711	110	1778	19
Siemens Trio Tim	2000	9.8	5800	103	2520	13
Siemens verio	1900	8.6	6000	97	1900	9.4
Philips Ingenia CX	6.4	3.1	4600	106	2000	20
Siemens Prisma	2000	9	5020	105	2190	9.2

### Image analysis

The tumor location, shape, signal intensity, enhancement pattern, maximum diameter, calcification, hemorrhage, peritumoral edema, relationship with the ventricle, and presence of “T2-FLAIR mismatch” sign (the tumor shows complete/near-complete hyperintensity on T2WI and demonstrates relatively hypointense signal with a hyperintense peripheral rim on FLAIR [5]) were independently analyzed by two imaging neuroradiologists. Discrepancy was resolved by consultation.

## Results

### Demographic data

Table 2 and Table 3 present the clinical and demographic characteristics of patients with PGNT. The onset age of PGNT was 3–43 years old. The most common symptom was epilepsy (27.8%, 10/36), followed by headache (19.4%, 7/36). In 36 cases of PGNT, except for 1 case where the brain stem mass was mostly removed and 1 case where biopsy was performed, all others were total resection

**Table 2:** Clinical and demographic characteristics of 36 patients with PGNT

Patient characteristics	
Age (years), mean ± SD	22.47 ± 11.23
< 18 years old, n (%)	13 (36.1)
≥ 18 years old, n (%)	23 (63.9)
Sex, n (%)	
Males	17 (47.2)
Females	19 (52.8)
Method for obtaining pathological results, n (%)	
Surgery	35 (97.2)
Biopsy	1 (2.8)
Ki-67 index	
Ki-67 < 5%	27 (75)
Ki-67 ≥ 5%	9 (25)

*Note:* Other symptoms including dizziness, limb weakness, blurred vision, convulsions, loss of consciousness, and postoperative reexamination of vascular malformations

**Table 3:** Main clinical symptoms of 36 patients with PGNT

Major symptoms or signs	Numbers, n (%)	Age (years)
Epilepsia(Generalized Onset)	8(22.2)	5–27
Epilepsia(tonic-clonic)	2(5.5)	15–23
Headache	7(19.4)	10–43
Headache and vomiting	5(13.8)	6–34
Headache and dizziness	4(11.1)	16–38
Headache and vomiting and hearing loss	1(2.8)	29
Headache and vomiting and epilepsia(generalized onset)	1(2.8)	18
Headache and unilateral mouth crooked and eye slanted	1(2.8)	25
Headache and vomiting and unilateral blurred vision	1(2.8)	35
Dizziness	1(2.8)	36
Unilateral limb weakness	1(2.8)	39
After surgery for intracranial vascular malformations	1(2.8)	3
Blurred vision	1(2.8)	21
Tinnitus and hearing loss and epilepsia(Generalized Onset)	1(2.8)	35
Unilateral tinnitus	1(2.8)	18

### Image feature

Table 4 shows PGNT imaging characteristics.

**Table 4:** PGNT image features

Index	Numbers
Location, n (%)	
Supratentorial	35(97.2)
Frontal lobe	11(31.4)
Temporal lobe	7(20)
Supratentorial ventricles	6(17.2)
Parietal lobe, occipital-parietal lobe	6(17.2)
Other location	5(14.2)
Subtentorial(brain stem)	1(2.8)
Ventricular relationship, n (%)	
intraventricular and periventricular location	29(80.6)
Distant	7(19.4%)
Size (maximum diameter), mean ± SD	42.47 mm ± 17.61 mm
Mass characteristics, n (%)	
Mixed cystic and solid	24(66.7)
Cystic with mural nodule	4(11.1)
Cystic	4(11.1)
Solid	4(11.1)
Septations, n (%)	25(69.4)
Peritumoral edema, n (%)	9(25)
Calcification, n (%)	9(25)
Intratumoral hemorrhage, n (%)	9(25)

### Mixed cystic and solid lesions

**Fig. 1:**
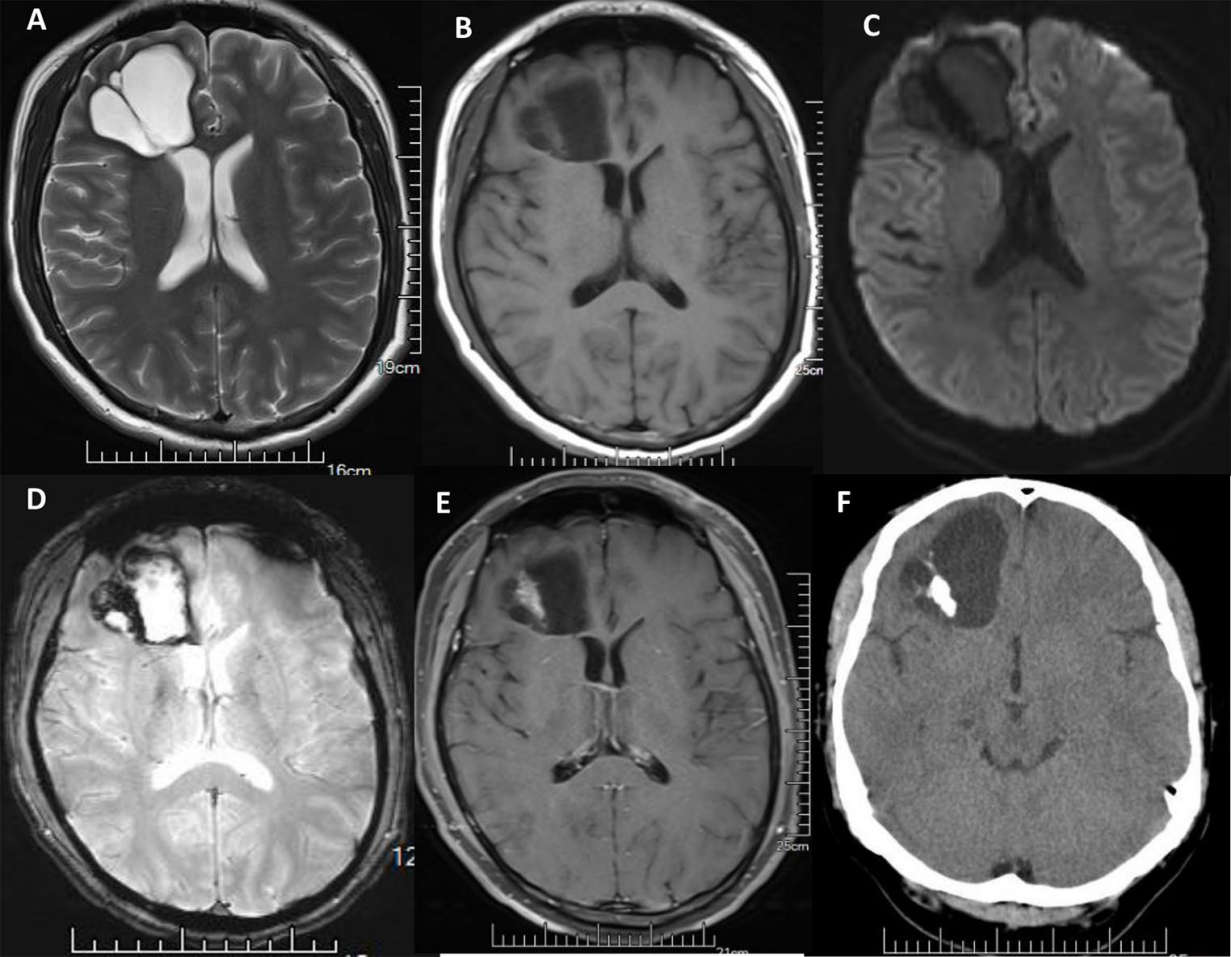
PGNT in the right frontal lobe of a 38-year-old male. **A**. Axial T2WI shows separation signals within PGNT. **B**. Axial T1WI shows mixed cystic and solid lesion. **C**. No diffusion restriction is observed in axial DWI. **D**: Axial SWI shows calcifications and hemorrhagic signals within the lesion. **E**. Axial enhancement shows heterogeneous enhancement in the solid part of the lesion. **F**. Axial CT shows spotty and patchy calcification within the lesion.

Twenty-four PGNTs were mixed cystic and solid lesions (Fig. 1).

Among them, eight were mainly cystic lesions, with few septations and small nodular shadows observed on T2WI, separations and small nodules with enhancement, and enhancement or non-enhancement of the cyst wall. Only one lesion showed no evidence of septation, while three cases had larger lesions with surrounding areas of mild edema.

Six lesions showed “loofah-like” changes on T2WI, with separated shadows, multiple nodular enhancement, enhancement or non-enhancement of the cyst wall. One case had small lesion and was surrounded by areas of mild edema.

Four tumors exhibited irregular tumor morphology, with partial concavity at the lesion edges accompanied by cystic lesions of varying sizes and septations and heterogeneous enhancement of the solid part; three tumors had patchy edema along the lesion edge.

There were two mixed cystic and solid lesions with solid components with honeycomb-like enhancement, and predominantly on one side, within the lesion, there were scattered and patchy calcifications. The edges of the lesion were accompanied by large cysts and septations.

Two mixed cystic and solid lesions showed honeycomb-like changes on T2WI, with small nodular enhancement visible at the enhancement edge.

Two mixed cystic and solid lesions showed local thickening of the cyst wall and heterogeneous enhancement, one of which did not show any septations and had surrounding small edema.

### Cystic lesions with mural nodules

Four PGNTs were cystic lesions with mural nodules. One lesion showed noticeable uniform enhancement of the wall nodules, and three showed large cysts with small nodular changes, where the nodules exhibited honeycomb-like enhancement towards the inner side, with or without enhancement of the capsular wall. One lesion also presented with septations within the cystic lesion.

### Pure cystic lesions

**Fig. 2:**
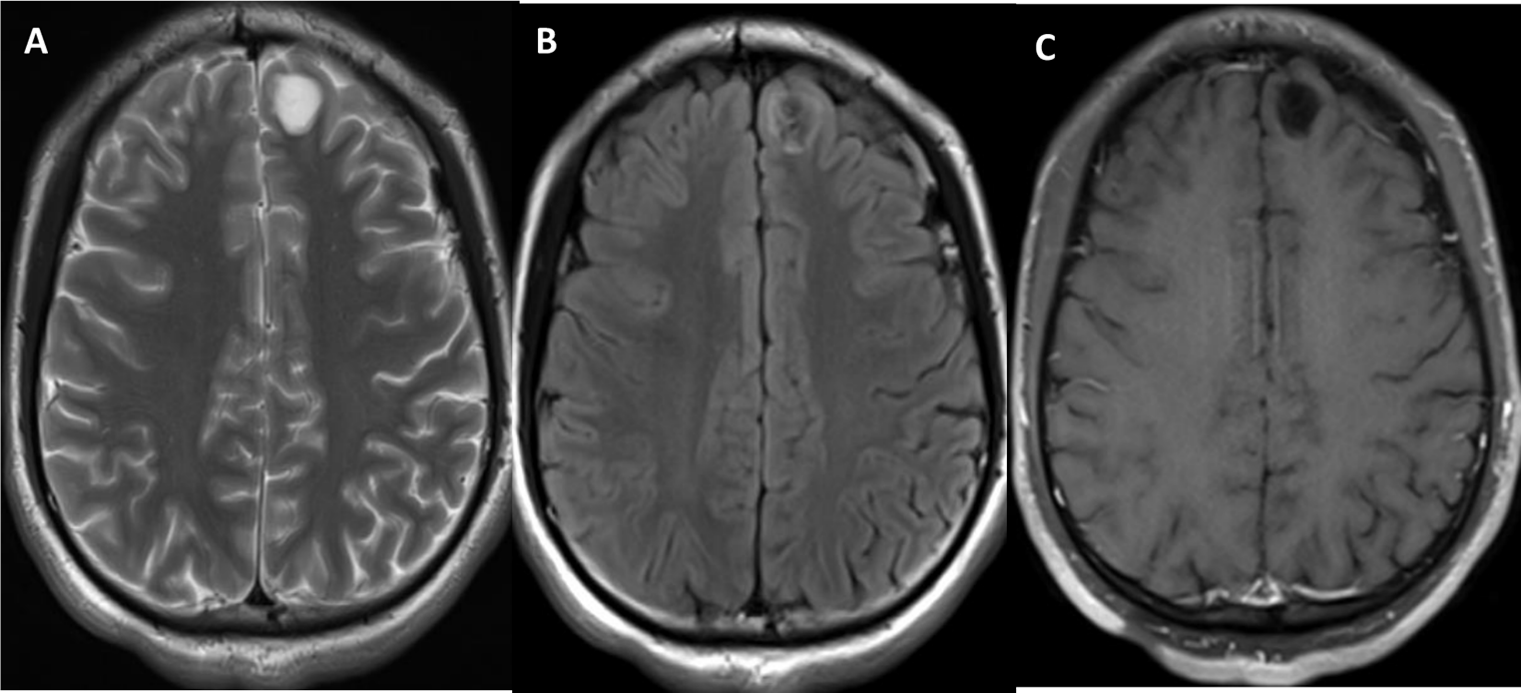
Pure cystic PGNT in the left frontal lobe of a 19-year-old male. (**A**) The lesion on axial T2WI shows uniform hyperintensity. (**B**) Axial T2-FLAIR shows “T2 FLAIR mismatch” sign. (**C**) Axial enhancement shows no enhancement of the lesion.

Four PGNTs presented as pure cystic lesions, all of which were small (Fig. 2). They exhibited a “T2-FLAIR mismatch” sign without enhancement, and no evidence of septation was observed.

### Pure solid lesions

Four PGNTs were pure solid lesions. Two showed noticeable heterogeneous enhancement with restricted DWI diffusion, accompanied by a septal shadow, and two showed noticeable uniform enhancement. One lesion had heterogeneous enhancement with small areas of edema along the margins of the lesion.

### Overall imaging

Of the 36 PGNT lesions, 25 showed septations within the lesions (Fig. 1A), and 11 showed no septations. There were nine lesions with edema, all of which were located outside the ventricle. Nine PGNT lesions were accompanied by spotty and plaque calcification (Fig. 1F); one was a solid lesion, and eight were mixed cystic and solid lesions. Nine mixed cystic and solid lesions had hemorrhage (Fig. 1D); the lesions were closely related to the ventricles. Two patients had hemosiderosis on the cerebral surface.

Twenty-six patients underwent DWI, of which 10 had a solid part of the lesion or cystic walls with restricted diffusion.

Twenty-seven patients had a Ki-67 index < 5%, and nine had a Ki-67 index ≥ 5% (three with solid lesions, four with mixed cystic and solid lesions, and two with cystic lesions with mural nodules). Among the 36 PGNT patients, synaptophysin was negative in only 1 patient with brain stem mass, while the others were positive. 34 patients were positive for GFAP, and 2 patients did not find GFAP results. 33 patients showed Olig-2 positivity, while 3 patients did not find Olig-2.

## Discussion

PGNT is a rare WHO grade I glial neuron tumor that accounts for 0.02% of all central nervous system tumors. The age of onset is 4–75 years old, the peak is of onset is 20–30 years old, and there is no significant sex predominance [2, 6]. The male-to-female ratio of patients in this group was 1:1.1, with an average age of 22.5 years, and the age of onset was 3–43 years, which is consistent with the literature. The minimum length diameter of the lesions was approximately 15 mm, the maximum length diameter was approximately 80 mm, and the median length was 45 mm, which is consistent with the average diameter of 138 PGNT lesions reported by Ahmed et al. [4].

PGNT usually presents with nonspecific clinical manifestations [7]. Headache, nausea, and vomiting caused by increased intracranial pressure are common initial symptoms, followed by seizures. Acute symptoms or focal neurological deficits rarely occur during the natural course of PGNT, possibly because it is a low-grade tumor with slow growth. Over time, its clinical symptoms are related to the progress of intracranial pressure or space-occupying effects [2, 8]. The initial symptoms of the patients in this group included epilepsy, headache, vomiting, dizziness, limb weakness, hearing symptoms, blurred vision, convulsion, stare blankly, etc., which occurred alone or in combination, consistent with previous reports.

PGNTs tend to occur in the supratentorial area, usually in the frontal and temporal lobes, and are closely related to the ventricles. Most PGNTs are located in the paraventricular white matter, and some of PGNTs may involve the ventricle. Sometimes, it occurs in the ventricles, but there are also reported cases in other locations, such as the cerebellum, pineal region, or brain stem [2, 8]. In our cohort, one tumor was located in the infratentorial brain stem, and 35 were found in the supratentorial region, including 11 in the frontal lobe, 7 in the temporal lobe, most of which were close to or involved the ventricles, 6 in the ventricle, and 1 in the pineal region. At present, the pathogenesis of PGNT is still unclear. Because of the close relationship between the tumor and ventricles, some studies have suggested that PGNT may originate from the germinal zone of the subependymal plate and that more superficial PGNT may arise from the secondary germinal layer [9]. The expression of stem cell markers within the tumor suggests a possible origin from neuroepithelial stem cells with biphenotypic differentiation [10].

PGNT imaging can be divided into four categories [11]: mixed cystic and solid mass, cystic mass with mural nodule, pure cystic mass, and pure solid mass. In this group, 66.7% (24/36) of the tumors were mixed cystic and solid mass, 11.1% (4/36) were cystic mass with mural nodule, 11.1% (4/36) were pure cystic mass, and 11.1% (4/36) were pure solid mass, most tumors did not have edema or had only mild peritumoral edema. In comparison, Park [11] summarized 76 cases of PGNT and found that cystic masses with mural nodules were the most common type. As that was a summary of multiple studies, there may be differences in tumor classification criteria, and further retrospective analysis with a large sample size is needed. Secondly, the number of tumors in this group was relatively small, and cystic mass with mural nodule is a type of mixed cystic and solid lesion.

PGNT may present with hemosiderin, calcification, septation, and superficial siderosis; the mass effect is mild, and mild perilesional edema is rare [6]. Only nine of the 36 lesions in our cohort were accompanied by edema. Septation was observed in 25 lesions, of which only one mixed cystic and solid lesion was not septated, which may be related to the obvious pseudopapillary structure of PGNT. The presence of prominent septations within the cystic component formed by long pseudopapillae traversing the cyst is a distinctive imaging feature [12]. Tavallaii et al. [2] mentioned that calcification can be seen in PGNT. Internal bleeding is an extremely rare radiological manifestation of these tumors; a hemorrhage in the form of chronic micro-bleeding leading to hemosiderosis has rarely been reported. Tan et al. [13] suggested that calcification is a common sign of mixed cystic and solid PGNT. Although only half of the patients in this cohort underwent CT or SWI examination simultaneously. Two patients had diffuse superficial siderosis, nine had mixed cystic and solid lesions, as well as a hemorrhage. Moreover, nine had calcification. Therefore, calcification and hemorrhage may be common signs of mixed cystic and solid PGNT. The pathogenesis of brain tumor-related hemorrhage mainly includes endothelial proliferation with subsequent obliteration of the lumen, thin-walled or poorly formed vessels, perivascular necrosis with subsequent loss of vessel support, and the presence of intratumoral arteriovenous fistulae [14]. Benzagmout et al. [15] described two cases of PGNT with hemorrhage at different periods. They believed that the pathogenesis of tumor hemorrhage included thin-walled blood vessels in the solid part or abnormally transparent blood vessels in the nipple area, so that PGNT could be accompanied by hemorrhage.

PGNT is a low-grade biphasic neoplasm with astrocyte and neuronal differentiation, with a prominent pseudopapillary architecture, composed of hyalinized vessels, and covered by single layer of small cuboidal glial cells [2, 7]. GFAP was positive in 34 patients in this group, and no GFAP results were found in 2 patients. Tanaka et al. [16] found another cellular component of PGNT, characterized by Olig-2 positivity, indicating a phenotype of oligodendrocytes. In this group, 33 patients were Olig-2 positive, 7 of which showed focal or sporadic positivity, and the Olig-2 results were not found in the other 3 patients. The average Ki-67 index of PGNT was 3.6%, with a Ki-67 < 5% in most cases, indicating low proliferative activity, absence, or low mitotic activity [4]. Most patients with PGNT have a good prognosis, and there are literature reports following patients for up to 26 years. Total tumor resection is the preferred treatment for PGNT, and although recurrence can sometimes occur after resection, in most cases the prognosis is good even if complete resection is not performed [8]. A Ki-67 ≤ 5% and maximum surgical resection are positive prognostic factors [17]. In our cohort, 27 patients with a Ki-67 < 5%, nine with a Ki-67 ≥ 5%. Four PGNT presented as pure cystic lesions, all of which were small, they exhibited a “T2-FLAIR mismatch” sign, and a Ki-67 < 5%. In 36 cases of PGNT, except for 1 case where the brain stem mass was mostly removed and 1 case where biopsy was performed, all others were total resection. However, patients with brain stem masses were not followed up. No recurrence was found among the nine patients who underwent postoperative review (the minimum postoperative review time was 6 months, and the longest was 24 months). Although PGNT is considered an indolent lesion, this tumor can be highly proliferative, which may indicate the possibility of transformation of PGNT to a more aggressive form [7]. The low frequency of PGNT and the description of only a few atypical cases may mask its occasional malignant potential [18]. Goethe et al. [19] found that a patient with PGNT had a Ki-67 index of 6–7%, and the tumor recurred 7 months after surgery with an increased Ki-67 index; the tumor recurred again 2 years after reoperation. Yadava et al. [20] reported a case of PGNT recurrence 9 years after surgery that was characterized by solid partial hyperperfusion, elevated Cho, intratumoral hemorrhage, and superficial siderosis. Carangelo et al. [21] reported their experience with a patient with PGNT with a Ki-67 index < 1% who relapsed 2 months after surgery. One of our patients had extensive flocculent lesions on the ventricle wall. A biopsy was performed, and a fistula was made at the bottom of the third ventricle. The biopsy indicated that the Ki-67 index was 2–5%. Multiple metastatic lesions in the brain were found 1 year and 4 months after the ostomy. Similarly, Bourekas et al. [18] reported a case of PGNT with anaplastic histological features that recurred three times after surgery and underwent resection. The patient developed multiple systemic metastases approximately 4.5 years later. These findings suggest that a high histological grade is more associated with central nervous system tumor metastasis, which may occur through vascular damage from surgery or radiation and via ventriculoperitoneal shunts. One of our patients developed metastasis, which may be related to surgical ostomy and the growth site.

This study has several limitations. First, the sample size was relatively small, mainly because of the low incidence of PGNT. Second, as this is a retrospective study, only some patients underwent CT or SWI examinations, which resulted in the inaccurate evaluation of the incidence of internal bleeding and calcification within the lesion. Third, the postoperative follow-up time was short; some patients did not undergo imaging follow-up examination after surgery. In conclusion, our results show that PGNTs mostly manifest as cystic mass with mural nodule or mixed cystic and solid mass in the white matter around the supratentorial ventricle and rarely occurs under the tentorium. Pure cystic lesions may exhibit the sign of “T2-FLAIR mismatch”. PGNT is seldom accompanied by edema and can be accompanied by calcification and hemorrhage. Pathological morphology and immunohistochemistry can help make a definite diagnosis.

## Data Availability

Not applicable.

## Abbreviations

MRI: Magnetic resonance imaging
CT: Computed tomography
TR: Time of repetition
TE: Time to echo
T1WI: TI-weighted imaging
T2WI: T2-weighted imaging
CE-T1WI: Contrast-enhanced TI-weighted imaging
SWI: Susceptibility-weighted imaging
DWI: Diffusion-weighted imaging
PGNT: Papillary glioneuronal tumor
T2-FLAIR mismatch: Presence of complete/near-complete hyperintense signal on T2WI and relatively hypointense signal on FLAIR except for a hyperintense peripheral rim
WHO: World Health Organization
